# A Flexible Congregate Meal Program for Older Adults in Hawaiʻi: A Quasi-Experimental Evaluation of Kūpuna U

**DOI:** 10.3390/nu17132106

**Published:** 2025-06-25

**Authors:** Jenny Jinyoung Lee, Nargis Sultana, Christy Nishita

**Affiliations:** Thompson School of Social Work & Public Health, University of Hawai‘i at Mānoa, 1960 East-West Road, Biomed C-105A, Honolulu, HI 96822, USAcnishita@hawaii.edu (C.N.)

**Keywords:** food insecurity, older adults, virtual nutrition program, congregate meals, home-delivered meals, senior nutrition, social isolation, program evaluation, COVID-19 adaptations

## Abstract

**Background:** Food insecurity and social isolation among older adults are pressing public health concerns that significantly impact physical and mental health outcomes. The COVID-19 pandemic exacerbated these challenges while forcing innovative adaptations to traditional congregate meal programs. **Objective**: This study examined the effectiveness of Kūpuna U, an alternative flexible congregate meal program comprising three models (virtual, hybrid, and traditional), in addressing food insecurity, loneliness, and self-rated health among older adults in Hawaiʻi. **Methods:** A quasi-experimental study with non-equivalent groups analyzed secondary program evaluation data. Participants (*N* = 270, follow-up *N* = 116) self-selected into virtual (grab-and-go meals + online activities), hybrid (grab-and-go meals + virtual and in-person activities), or traditional (in-person congregate meals + in-person activities) models. Food insecurity (6-item scale), loneliness (UCLA 3-item scale), and self-rated health (5-point scale) were measured at the baseline and 6-month follow-up. **Results:** The Virtual group showed significant improvements in food insecurity (baseline: 1.73 to follow-up: 0.04, *p* < 0.001) and self-rated health (baseline: 2.92 to follow-up: 3.72, *p* = 0.005). The Hybrid group demonstrated a significant increase in loneliness (baseline: 4.25 to follow-up: 5.00, *p* = 0.024). The Traditional group showed no significant changes in any outcome measures. Analysis of variance (ANOVA) revealed significant between-group differences for food insecurity (F = 9.047, *p* < 0.001) and self-rated health (F = 5.814, *p* = 0.004) change scores. **Conclusions:** The Virtual model demonstrated a superior effectiveness in improving food security and self-rated health outcomes. However, self-selection bias limits causal inferences. These findings suggest that flexible, technology-enhanced nutrition programs may effectively serve older adults with mobility or transportation barriers while maintaining program benefits.

## 1. Introduction

Food insecurity and social isolation among older adults are pressing public health concerns that significantly impact the overall well-being of this vulnerable population. The U.S. Department of Agriculture describes food insecurity as having restricted or unpredictable access to sufficient, nutritious, and safe food or lacking the means to obtain it in a socially acceptable manner [1]. In 2021, approximately 8.7% of Americans aged 60 and older (6.9 million) experienced food insecurity [2]. Older adults facing food insecurity are at an increased risk of adverse mental and physical health outcomes. This represents a concerning increase from pre-pandemic levels, as older adults continue to face unique challenges accessing adequate nutrition [3,4].

Food insecurity in older adults is associated with numerous adverse health outcomes, including an increased prevalence of chronic conditions, medication non-adherence, functional limitations, and compromised mental health [5]. Gundersen and Ziliak’s comprehensive review demonstrated that food-insecure older adults have significantly higher rates of depression, diabetes, and congestive heart failure compared to their food-secure counterparts [6]. Beyond physical health impacts, recent research indicates that food insecurity contributes to accelerated cognitive decline and increased healthcare utilization and costs [7,8]. Approximately USD 52.9 billion in healthcare expenditures annually can be attributed to food insecurity-related conditions among adults [9].

Furthermore, social isolation in older adults has been linked to a higher likelihood of depression, cognitive decline, and increased mortality risk [10,11]. The health impacts of social isolation are comparable to those associated with other well-established risk factors such as smoking, obesity, and physical inactivity [12]. During the COVID-19 pandemic, enforced physical distancing measures, while necessary for infection control, exacerbated isolation among older adults [13,14], with some studies reporting deterioration in mental health outcomes specifically linked to decreased social engagement [14].

Community-based nutrition programs, such as the congregate meal program (CMP), play a crucial role in mitigating these issues by providing access to nutritious meals and fostering social engagement. The CMP is a key initiative under the Older Americans Act (OAA) designed to enhance the nutritional well-being of older adults while also promoting social connectedness [15]. The traditional CMP typically operate in communal settings such as senior centers, churches, and community centers, where older adults can gather for meals and participate in social and educational activities [15]. Research indicates that these programs provide participants with approximately 40–50% of their daily nutritional needs and significantly reduce hospitalization rates [16].

The COVID-19 pandemic posed unprecedented challenges to these programs, as in-person gatherings became high-risk activities for older adults, who were particularly vulnerable to severe illness [17]. In March 2020, traditional congregate sites were forced to close, necessitating innovative approaches to serve vulnerable older adults while minimizing health risks [18]. The Administration for Community Living issued waivers allowing nutrition programs to adapt service delivery methods and reallocate funding between congregate and home-delivered meal programs [19].

In response to these challenges, Lanakila Meals on Wheels in Honolulu County launched the Kūpuna U, an alternative flexible CMP during the COVID-19 pandemic. Kūpuna is the Hawaiʻian word for elders, and the ‘U’ in Kūpuna U stands for “University,” reflecting the program’s educational platform, where participants accessed activities and education virtually. While COVID-19 created immediate challenges, it also accelerated the need to address pre-existing issues in traditional congregate programs, which were already experiencing declining participation nationwide. This innovative approach directly addresses the three core intents of the OAA Senior Nutrition Program: reducing hunger and food insecurity, promoting socialization, and enhancing health and well-being, particularly for vulnerable older adults with the greatest social and economic need.

Kūpuna U leveraged funding flexibility between Congregate Nutrition Services (C1) and Home-Delivered Nutrition Services (C2) [19], offering grab-and-go options or home delivery combined with virtual and in-person educational and socialization activities. The program was designed with replicability and sustainability in mind, utilizing existing OAA Title IIIC-1 funding mechanisms while modernizing service delivery through technology and partnerships.

A unique feature of the program was its partnership with the Kūpuna Food Security Coalition (KFSC), a network of over 40 organizations working collaboratively to address food insecurity among older adults in Hawai’i, which emerged in response to COVID-19 [20]. This coalition structure aligns with emerging evidence suggesting that multi-sectoral partnerships can enhance the reach and effectiveness of nutrition interventions beyond just meal provision [21,22]. While Lanakila Meals on Wheels maintained responsibility for meal services, the KFSC partnership significantly expanded the educational and socialization components of the program [23]. Research by Hoey et al. and Lee et al. examining collective impact models for food systems change found that such partnerships can improve coordination, reduce the duplication of services, and better leverage community resources to address comprehensive needs [20,22]. The coalition model employed by Kūpuna U represents an innovative approach to enhancing the non-meal aspects of senior nutrition programs through systems-level collaboration, creating a more holistic service model that addresses both nutritional needs and social engagement.

**Figure 1:**
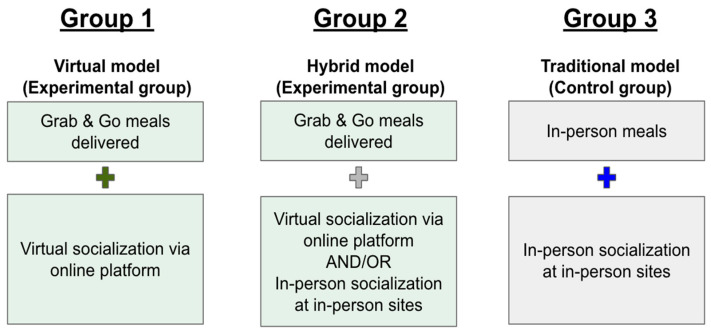
Three-groups model of Kūpuna U.

The Kūpuna U program employed three different service models: (1) a Virtual model providing grab-and-go meals with online socialization opportunities; (2) a Hybrid model combining grab-and-go meals with both virtual and in-person socialization; and (3) a Traditional model offering in-person congregate meals with in-person socialization (Figure 1). This innovative approach increased the reach of the program and also allowed for comparison between traditional and alternative service delivery methods. This research utilized secondary data analysis of Kūpuna U program data that was originally collected for reporting to the funder, the Administration for Community Living. The evaluation was designed to validate program effectiveness through three primary objectives: (1) verify positive impacts on participants’ food security, social connectedness, and well-being; (2) evaluate the effectiveness of collaborative strategies with community partners; and (3) examine systems-level efficiency, policy impacts, and sustainability of the program in delivering alternative congregate meal services.

This research paper focuses specifically on the first objective, examining the effectiveness of the three Kūpuna U models in addressing food insecurity, social isolation, and improving self-rated health among participating older adults. The study hypothesis was that all three models would show improvements in these outcomes over time, with the Virtual and Hybrid models demonstrating comparable effectiveness to the Traditional model. The findings will contribute to understanding the replicability and sustainability of virtual congregate meal programs in addressing the needs of older adults, particularly in times of crisis, and inform future policy decisions regarding flexible funding mechanisms for senior nutrition programs [24].

## 2. Methods

### 2.1. Study Design

A quasi-experimental study design was used to evaluate the effectiveness of the intervention. Data were collected from participants before and after the intervention across the three groups based on their self-selected engagement model. The Virtual and Hybrid models were considered experimental groups, while the Traditional model served as the control group (Figure 1). Data collection occurred between September 2021 and March 2023, with baseline surveys administered upon enrollment and follow-up surveys conducted at six-month intervals.

### 2.2. Participants

Participants (age 60+) were recruited for the Lanakila Meals on Wheels program through community partners such as a local senior center, senior housing, and local churches in Honolulu County. During registration, participants self-selected into their preferred program model (virtual, hybrid, or traditional) based on their needs and preferences. Participants in the Virtual model accessed daily recreational content through the Kūpuna U online platform and participated in various activities virtually Monday through Friday. Participants in the Hybrid model had the flexibility to engage in various in-person and online programs at two different sites (Senior Center and Senior Housing). Participants in the Traditional model engaged in activities and received hot meals at three different sites: a public library, a church, and a senior center.

All participants completed a baseline survey upon enrollment in the program, followed by two semi-annual follow-up surveys at six-month intervals. Baseline data were collected when participants joined the program using a structured survey. The survey gathered demographic details, including age, gender, ethnicity, educational status, living arrangements, and information related to food security, loneliness, and self-rated health status. These last three measures—food security, loneliness, and self-rated health—served as key evaluation criteria for assessing nutrition, socialization, and overall well-being.

### 2.3. Measurement Tools

Food security was assessed using the Six-Item Short Form of the U.S. Household Food Security Survey Module [25]. This validated tool measures participants’ access to adequate and nutritious food (scores range from 0 to 6), with higher scores indicating greater food insecurity. Loneliness was measured using the UCLA 3-Item Loneliness Scale [26], which assesses feelings of companionship, being left out, and isolation. Scores range from 0 to 9, with higher scores indicating greater loneliness. Self-rated health was evaluated using a 5-point Likert scale question adapted from the Behavioral Risk Factor Surveillance System tool [27]. Participants rated their health from excellent (5 points) to poor (1 point), with higher scores indicating better perceived health status.

### 2.4. Data Analysis

Statistical analysis was conducted using IBM’s Statistical Package (IBM SPSS V29.0) for the Social Sciences. Descriptive statistics were generated to characterize participants’ sociodemographic profiles. Baseline demographic characteristics were examined descriptively to identify potential differences between the three program groups.

Statistical assumptions for parametric tests (*t*-test and analysis of variance (ANOVA)) were assessed and considered acceptable for the analyses conducted. Within-group changes were evaluated using paired-sample *t*-tests comparing baseline to follow-up scores for food insecurity, loneliness, and self-rated health. Between-group differences in how scores changed were analyzed utilizing one-way ANOVA, with Tukey’s HSD post hoc tests for pairwise comparisons when significant main effects were detected. Effect sizes were calculated using R^2^ to assess practical significance and facilitate cumulative science [28].

Missing data were addressed using pairwise deletion to maximize the available data for analysis while maintaining statistical integrity. Statistical significance was set at *p* < 0.05.

## 3. Results

### 3.1. Demographic and Social Characteristics

Total participants (*N* = 270) were distributed across three delivery models: Virtual (*N* = 39), Hybrid (*N* = 154), and Traditional (*N* = 77). Of these, 116 participants completed both the baseline and second (annual) follow-up survey, representing a 43% retention rate. These participants were distributed across the Virtual (*N* = 27), Hybrid (*N* = 74), and Traditional (*N* = 15) delivery models (Table 1).

Female participants comprised more than 75% of baseline and follow-up surveys. In both surveys, most participants were between the ages of 65 and 84. Most participants in both surveys had some college education or an associate degree. Around 50% of baseline and follow-up survey participants lived alone.

Examination of baseline demographic and social characteristics (Table 1) revealed notable differences between the three groups. The Virtual group had a higher proportion of participants with bachelor’s degrees or higher (67%) compared to the Hybrid (27%) and Traditional groups (33%). Living arrangements also differed substantially, with the Hybrid group having the highest proportion of participants living alone (58%) compared to the Virtual (30%) and Traditional (20%) groups. Additionally, marital status patterns varied, with the Virtual group having the highest proportion of married participants (59%) compared to the Hybrid group (26%), while the Traditional group had an intermediate proportion (73%). The gender distribution was similar across the Virtual and Hybrid groups (approximately 75% female), while the Traditional group was entirely female (100%).

### 3.2. Food Insecurity Outcomes

Within-group analysis revealed a statistically significant improvement in the Virtual group (*p* < 0.001), marginal improvement in the Hybrid group (*p* = 0.07), and no change in the Traditional group (Table 2, additional detail in Appendix A). Between-group ANOVA revealed statistically significant differences in change scores (F(2,108) = 9.047, *p* < 0.001) though the effect size is low (R^2^ = 0.143). Post hoc analyses using Tukey’s HSD indicated that the Virtual group achieved a significantly greater improvement than both the Hybrid group (*p* < 0.001) and Traditional group (*p* = 0.003). No significant difference was found between the Hybrid and Traditional groups (*p* = 0.788).

### 3.3. Loneliness Outcomes

The Hybrid group showed a statistically significant increase in loneliness scores (*p* = 0.024), while the Traditional (*p* = 0.582) and Virtual groups (*p* = 0.357) showed no significant changes (Table 2). However, between-group ANOVA revealed no statistically significant differences in change scores (F(2,95) = 2.779, *p* = 0.067, R^2^ = 0.055). Post hoc analyses confirmed no significant pairwise differences between any groups (all *p* > 0.05).

### 3.4. Self-Rated Health Outcomes

The Virtual group demonstrated a statistically significant improvement in self-rated health (*p* = 0.005), while the Hybrid (*p* = 0.113) and Traditional groups (*p* = 0.424) showed no significant changes (Table 2). Between-group ANOVA revealed statistically significant differences in change scores (F(2,99) = 5.814, *p* = 0.004) though the effect size is low (R^2^ = 0.105). Post hoc analyses using Tukey’s HSD indicated that the Virtual group improved significantly more than the Hybrid group (*p* = 0.003). No significant differences were found between the Virtual and Traditional groups (*p* = 0.383) or between the Hybrid and Traditional groups (*p* = 0.414).

## 4. Discussion

This study evaluated the impact of Kūpuna U, an innovative alternative congregate meal program for older adults developed in response to the COVID-19 pandemic in Hawaiʻi. The findings revealed that the Virtual model demonstrated modestly superior outcomes for both food insecurity and self-rated health compared to hybrid and traditional approaches. The research compared three delivery models—Virtual, Hybrid, and Traditional—to determine their effectiveness in addressing older adults’ nutritional well-being and social connectedness by measuring the participants’ food insecurity, social isolation, and self-rated health. By examining 116 completed baseline and follow-up questionnaires, this study provided insights into how different delivery methods affect key outcomes for older adults.

### 4.1. Food Insecurity 

The Virtual model substantially reduced food insecurity scores. This improvement suggests that the combination of technology-based engagement with home-delivered meals effectively addressed food access challenges for older adults. Changes in food insecurity associated with the Hybrid model did not reach statistical significance, while the Traditional group maintained stable scores, potentially indicating that participants in this group had consistently adequate food security throughout the study period.

These results align with the emerging literature suggesting that technology-mediated nutrition interventions can enhance access to nutrition services, particularly for older adults with mobility limitations or transportation barriers [29]. Similar to findings reported by Lee et al. [23], the Virtual model may reduce participation barriers by eliminating the need for a physical presence at meal sites, potentially reaching older adults whom traditional congregate models previously underserved.

By removing transportation barriers, the Virtual model appears to enhance food security benefits for previously marginalized participants. This is consistent with research by Marx et al., demonstrating that virtual nutrition interventions combined with meal delivery services can significantly improve nutritional outcomes among homebound older adults while reducing barriers to accessing nutrition care [30].

The success of the Virtual model suggests that allowing flexibility in OAA Title IIIC-1 funding to support virtual congregate meal formats could extend nutritional benefits to previously underserved populations. As Lee et al. [23] noted in their focus group findings, participants appreciated having access to nutritious meals without the challenges of transportation, which was especially valuable for those with mobility limitations.

### 4.2. Loneliness and Social Connectedness

Contrary to expectations, the participants in the Hybrid model showed increased loneliness, while the participants in the Virtual and Traditional models did not show significant changes. A detailed examination of participant characteristics reveals important patterns that help explain these findings.

Participants at the senior housing location within the Hybrid group had distinctive characteristics: 80% lived alone (24 out of 30), compared to 50% at the senior center location, and 53% were in the 80+ age categories, versus only 16% at the senior center. Researchers observed that these participants frequently experienced mobility limitations not captured in the quantitative data. Among these 24 participants living alone, 83% either showed no change or an increase in loneliness scores.

This unexpected finding aligns with research by Cornwell and Waite [10], who found that social disconnectedness and perceived isolation can have different effects on older adults depending on their living situations and existing social networks. The National Academies of Sciences, Engineering, and Medicine [11] comprehensive report on social isolation emphasizes that interventions must be tailored to individual circumstances to be effective.

While the ANOVA did not detect statistically significant differences across the three program models, this result should be interpreted cautiously given the small sample sizes. The subgroup analysis suggests that participants’ responses to different program modalities were likely influenced by their personal circumstances rather than the program model alone. Kotwal et al. [14] found similar variations in loneliness responses during COVID-19 shelter-in-place orders, particularly among older adults living alone.

The virtual program appeared particularly beneficial for older adults living alone with mobility challenges, possibly by providing consistent social connection without transportation stress. This aligns with findings from Lee et al. [23], where focus group participants reported that the program enhanced their social engagement and learning experiences.

These findings highlight how program effectiveness may be moderated by the intersection of age, living situation, and mobility status rather than by a single factor. Future research with larger samples should investigate how these characteristics interact with the program modality to affect loneliness outcomes in older adults.

### 4.3. Self-Rated Health

Self-rated health improved among participants in the Virtual model, indicating enhanced perceptions of overall wellbeing. This improvement suggests potential benefits from the combination of online activities/classes with nutritional support. The Hybrid and Traditional models did not demonstrate any significant change in self-rated health.

Similar trends emerged with self-rated health as with food insecurity, with the Virtual group showing improvement, while the Hybrid and Traditional groups showed no statistically significant changes. The marked difference between the Hybrid and Virtual groups suggests that the intervention format interacts with participant characteristics to influence self-rated health outcomes, similar to what we observed with loneliness. As with social connectedness, the effectiveness of program models for improving self-rated health likely depends on individual circumstances including the living situation, mobility limitations, and existing support networks.

The improvement in self-rated health among virtual program participants may be attributed to several factors. Access to consistent nutritional support through home-delivered meals and health education components delivered virtually may have positively influenced participants’ perception of their health status. This aligns with research by Lloyd and Wellman [31] and Thomas and Mor [24] demonstrating that nutrition programs can impact health perceptions beyond just nutritional outcomes.

Virtual programming also allows for greater personalization and accessibility of health information, potentially increasing participants’ sense of self-efficacy in managing their health. LoBuono and Milovich [29] found that technology-based nutrition interventions can effectively deliver tailored health information to older adults, contributing to improved self-perceptions of health. This suggests that the Virtual model’s ability to deliver tailored content may contribute to its effectiveness in improving self-rated health.

### 4.4. Comparative Program Effectiveness

When comparing the relative impact between the three program models, the ANOVA results consistently identified the Virtual model as the most effective approach for improving food security and self-rated health. This finding has significant implications for the replicability and sustainability of alternative congregate meal programs nationwide. The Virtual model may present a cost-effective alternative that requires less physical infrastructure while potentially serving more participants, particularly those with mobility limitations or transportation barriers who have historically been excluded from traditional congregate settings.

Implementing this effective Virtual model, however, requires significant collaborative infrastructure. The success of Kūpuna U depended on the Kūpuna Food Security Coalition (KFSC), a network of over 40 organizations that provided diverse educational and socialization content [20]. Organizations considering replication should anticipate developing formal partnerships with community entities, establishing accessible IT infrastructure, providing technology training, and implementing flexible meal delivery systems. While potentially more cost-effective long-term, organizations should prepare for initial investments in technology, staff training, and partnership coordination. Creating a similar coalition requires dedicated coordination staff and regular communication mechanisms to maximize resource sharing and minimize service duplication.

The Traditional model group showed no statistically significant changes in any of the measured outcomes (food insecurity, loneliness, or self-rated health). Participants’ scores remained relatively stable from baseline to follow-up. While traditional congregate meal programs have demonstrated benefits over decades of implementation [32,33], the current findings suggest that virtual adaptations may enhance program reach and effectiveness, particularly for older adults who face barriers to participating in site-based programs, thus better serving those with the greatest social and economic need as mandated by the OAA.

These comparative findings suggest that program administrators should consider tailoring delivery approaches—such as incorporating virtual and hybrid service models—to better meet participant needs and preferences, rather than relying on a one-size-fits-all solution. Additionally, the superior performance of the Virtual model in certain domains suggests that policymakers should consider permanent flexibility in OAA funding regulations to allow virtual congregate meal programming as a recognized service delivery method. This aligns with recommendations from previous studies [31] advocating for innovation and flexibility in OAA program implementation.

### 4.5. Limitations

Several limitations should be considered when interpreting these findings. First, an important limitation was the self-selection bias inherent in the study design. Participants chose their engagement model based on personal preferences, needs, and circumstances rather than random assignment. Examination of baseline demographics revealed notable differences between groups, including educational attainment, living arrangements, and marital status, suggesting that different types of participants were drawn to different program models. For example, participants choosing the Virtual model had higher education levels and were more likely to be married, while those selecting the Hybrid model were more likely to live alone. These systematic differences between groups limit the ability to determine whether observed differences in outcomes were due to the program models themselves or due to pre-existing characteristics of participants who chose different models. Second, there was a small sample size for the Traditional group (*N* = 15) compared to the Hybrid (*N* = 74) and Virtual (*N* = 27) groups. This disparity reflected real-world implementation patterns during the COVID-19 pandemic, where traditional congregate dining was less available and less preferred by older adults due to health concerns. The small Traditional group sample substantially limits the statistical power for detecting meaningful changes and reduces the precision of effect size estimates. Consequently, null findings for the Traditional group should be interpreted cautiously, as this study may have been underpowered to detect clinically meaningful changes. Third, this study relied on self-reported measures subject to recall bias and social desirability effects.

An important limitation was the lack of data on participants’ prior experience with congregate meal programs before joining Kūpuna U. This missing baseline information made it difficult to determine whether observed outcomes were influenced by previous congregate meal participation. Additionally, staff sometimes noted participant improvements that were not reflected in self-reported survey responses, suggesting potential measurement limitations or response biases.

Cultural factors may have affected response patterns, particularly for food insecurity measures. Program staff noted that some participants, especially from certain cultural backgrounds, expressed a reluctance to acknowledge food insecurity due to perceived shame or stigma. This social desirability bias might have led to underreporting of food insecurity, potentially masking the program’s full impact on this outcome measure.

The subjective nature of the measurement tools used presents another limitation. Self-reported outcomes are inherently influenced by participants’ perceptions, cultural backgrounds, and willingness to disclose certain information. These subjective measurements, while valuable, may not fully capture objective changes in nutrition status, social engagement, or health outcomes.

As noted in the data analysis presentation, expanding the program and improving data collection completeness would strengthen future evaluations. Additionally, tracking participant characteristics such as technology proficiency, prior social engagement patterns, and baseline health status would help identify for whom each delivery model is most effective.

The COVID-19 pandemic context in which this program was implemented represents a unique circumstance that may limit generalizability to non-emergency periods. However, the innovations developed during this period provide valuable insights that can inform ongoing program development and optimization.

## 5. Conclusions

This evaluation of the Kūpuna U program reveals a paradigm shift in how congregate meal programs can effectively serve older adults. The Virtual model’s superior performance in improving food security and self-rated health challenges long-held assumptions that in-person gatherings are essential for program effectiveness. This finding has significant policy implications: Title IIIC-1 funding flexibility to support virtual delivery formats could extend nutritional benefits to previously underserved populations, particularly those with mobility limitations, transportation barriers, or who live in isolated areas. The differential impacts observed across participant groups underscore the importance of person-centered approaches that consider individual circumstances rather than one-size-fits-all solutions. As the aging services network seeks to modernize nutrition programs while using their limited funding effectively, the Kūpuna U model offers a replicable and sustainable template that can be adapted across diverse geographic and demographic contexts. The pandemic-driven innovations tested in this study offer valuable insights for creating more accessible, effective, and inclusive nutrition services that can enhance food security and well-being for all older adults, regardless of their physical ability to attend congregate sites.

## Figures and Tables

**Table 1:** Participants’ demographic information.

		Baseline								Follow-Up							
		All		Hybrid		Traditional		Virtual		All		Hybrid		Traditional		Virtual	
		*N*	%	*N*	%	*N*	%	*N*	%	*N*	%	*N*	%	*N*	%	*N*	%
		270		154	57	77	29	39	14	116		74	64	15	13	27	23
Demographics																	
Gender																	
	Female	207	76.66	110	71.43	68	88.31	29	74.36	92	79.31	57	77.03	15	100	20	74.07
	Male	62	23	43	27.92	9	11.69	10	25.64	24	20.69	17	22.97		0	7	25.93
	Other	1	0.37	1	0.65	-	-	-	-								
Age																	
	60 to 64	17	6.30	8	5.19	8	10.39	1	2.56	6	5.17	1	1.35	4	26.67	1	3.7
	65 to 69	45	16.67	23	14.94	16	20.78	6	15.38	17	14.66	10	13.51	3	20	4	14.81
	70 to 74	51	18.89	37	24.03	9	11.69	5	12.82	19	16.38	14	18.92	2	13.33	3	11.11
	75 to 79	61	22.59	32	20.78	17	22.08	12	30.77	27	23.28	15	20.27	5	33.33	7	25.93
	80 to 84	50	18.52	28	18.18	13	16.88	9	23.08	25	21.55	17	22.97	1	6.67	7	25.93
	85+	31	11.48	15	9.74	12	15.58	4	10.26	13	11.21	10	13.51		0	3	11.11
	Unknown	15	5.56	11	7.14	2	2.6	2	5.13	9	7.76	7	9.46		0	2	7.41
Race																	
	Black	9	3.33	5	3.25	2	2.6	2	5.13	3	2.59	1	1.35		0	2	7.41
	Chinese	27	10	8	5.19	8	10.39	11	28.21	15	12.93	4	5.41	2	13.33	9	33.33
	Filipino	9	3.33	5	3.25	4	5.19	-	-	3	2.59	3	4.05		0		0
	Japanese	95	35.19	38	24.68	38	49.35	19	48.72	37	31.9	18	24.32	8	53.33	11	40.74
	Korean	14	5.19	14	9.09	-	-	-	-	5	4.31	5	6.76		0		0
	Other Asian	49	18.15	40	25.97	8	10.39	1	2.56	33	28.45	29	39.19	3	20	1	3.7
	Native Hawaiʻian	15	5.56	6	3.9	8	10.39	1	2.56	2	1.72	2	2.7		0		0
	Pacific Islander	4	1.48	3	1.95	1	1.3	-	-	1	0.86	1	1.35		0		0
	White	44	16.3	31	20.13	8	10.39	5	12.82	16	13.79	10	13.51	2	13.33	4	14.81
	Hispanic	4	1.48	4	2.6	-	-	-	-	1	0.86	1	1.35		0		0
Education level																	
	Did not complete high school	14	5	10	6	4	5	-	-	5	4	5	7		0		0
	High school graduate	62	23	43	28	16	21	3	8	24	21	22	30	1	7	1	4
	Some college or associate degree	98	36	61	40	25	32	12	31	41	35	25	34	8	53	8	30
	Bachelor’s degree or higher	89	33	37	24	28	36	24	62	43	37	20	27	5	33	18	67
	Unknown	7	3	3	2	4	5	-	-	3	3	2	3	1	7		0
Marital status										116							
	Married	96	35.56	40	25.97	34	44.16	22	56.41	46	39.66	19	25.68	11	73.33	16	59.26
	Single	98	36.3	75	48.7	12	15.58	11	28.21	45	38.79	36	48.65	1	6.67	8	29.63
	Widowed	63	23.33	32	20.78	25	32.47	6	15.38	22	18.97	17	22.97	2	13.33	3	11.11
	Other	13	4.81	7	4.55	6	7.79	-	-	3	2.59	2	2.7	1	6.67	-	-
Living arrangement																	
	Living alone	137	50.74	94	61.04	30	38.96	13	33.33	54	46.55	43	58.11	3	20	8	29.63
	Living with a Spouse	87	32.22	35	22.73	29	37.66	23	58.97	42	36.21	16	21.62	10	66.67	16	59.26
	Living with other family members	37	13.7	19	12.34	16	20.78	2	5.13	15	12.93	11	14.86	2	13.33	2	7.41
	Living with others	9	3.33	6	3.9	2	2.6	1	2.56	5	4.31	4	5.41		0	1	3.7

Note: *N* = number of participants, % = percent of participants.

**Table 2:** Changes in average score.

Groups	Participants	Baseline	Follow-Up	*p* Value
	Food Insecurity (*N* = 111)			
Hybrid	71	0.77	0.48	0.07
Traditional	14	No differences between the baseline andfollow-up survey		
Virtual	26	1.73	0.04	<0.001
	Loneliness (*N* = 98)			
Hybrid	60	4.25	5	0.024
Traditional	15	3.8	3.67	0.582
Virtual	23	4.3	3.83	0.357
	Self-Rated Health (*N* = 101)			
Hybrid	62	3.18	2.87	0.113
Traditional	15	3.6	3.8	0.424
Virtual	24	2.92	3.75	0.005

## Data Availability

The original contributions presented in this study are included in the article. Further inquiries can be directed to the corresponding author.

## Abbreviations

COVID-19	Coronavirus Disease 2019
CMP	Congregate Meal Program
OAA	Older Americans Act
KFSC	Kūpuna Food Security Coalition
ANOVA	Analysis of Variance

## Appendix A

**Table A1:** Tests of between-subjects effects for food insecurity score.

Dependent Variable: Base to Follow-Up					
Source	Type III Sum of Squares	df	Mean Square	F	Sig.
Corrected Model	42.610 a	2	21.305	9.047	<0.001
Intercept	31.881	1	31.881	13.538	<0.001
Group	42.610	2	21.305	9.047	<0.001
Error	254.327	108	2.355		
Total	335.000	111			
Corrected Total	296.937	110			

a: R Squared = 0.143 (Adjusted R Squared = 0.128).

**Table A2:** Post hoc test for food insecurity score.

Multiple Comparisons							
	(I) Groups	(J) Groups	Mean Difference (I-J)	Std. Error	Sig.	95% Confidence Interval	
						Lower Bound	Upper Bound
Tukey HSD	Hybrid	Traditional	−0.2958	0.44875	0.788	−1.3622	0.7707
		Virtual	1.3965 *	0.35177	<0.001	0.5606	2.2325
	Traditional	Hybrid	0.2958	0.44875	0.788	−0.7707	1.3622
		Virtual	1.6923 *	0.5087	0.003	0.4834	2.9012
	Virtual	Hybrid	−1.3965 *	0.35177	<0.001	−2.2325	−0.5606
		Traditional	−1.6923 *	0.5087	0.003	−2.9012	−0.4834
Scheffe	Hybrid	Traditional	−0.2958	0.44875	0.805	−1.4096	0.8181
		Virtual	1.3965 *	0.35177	<0.001	0.5234	2.2696
	Traditional	Hybrid	0.2958	0.44875	0.805	−0.8181	1.4096
		Virtual	1.6923 *	0.5087	0.005	0.4297	2.955
	Virtual	Hybrid	−1.3965 *	0.35177	<0.001	−2.2696	−0.5234
		Traditional	−1.6923 *	0.5087	0.005	−2.955	−0.4297

Based on observed means. The error term is mean square (error) = 2.355. * The mean difference is significant at the 0.05 level.

**Table A3:** Tests of between-subjects effects for loneliness score.

Dependent Variable: Base to Follow-Up					
Source	Type III Sum of Squares	df	Mean Square	F	Sig.
Corrected Model	28.829 a	2	14.414	2.779	0.067
Intercept	0.151	1	0.151	0.029	0.865
Groups	28.829	2	14.414	2.779	0.067
Error	492.722	95	5.187		
Total	532	98			
Corrected Total	521.551	97			

a: R Squared = 0.055 (Adjusted R Squared = 0.035).

**Table A4:** Tests of between-subjects effects for self-rated health score.

Dependent Variable: Base to Follow-Up					
Source	Type III Sum of Squares	df	Mean Square	F	Sig.
Corrected Model	22.266 a	2	11.133	5.814	0.004
Intercept	3.917	1	3.917	2.046	0.156
Groups	22.266	2	11.133	5.814	0.004
Error	189.577	99	1.915		
Total	212	102			
Corrected Total	211.843	101			

a: R Squared = 0.105 (Adjusted R Squared = 0.087).

**Table A5:** Post hoc test for self-rated health score.

Multiple Comparisons							
Dependent Variable: Base to Follow-Up							
	(I) Groups	(J) Groups	Mean Difference (I-J)	Std. Error	Sig.	95% Confidence Interval	
						Lower Bound	Upper Bound
Tukey HSD	Hybrid	Traditional	−0.5065	0.39818	0.414	−1.4539	0.441
		Virtual	−1.1065 *	0.32785	0.003	−1.8866	−0.3264
	Traditional	Hybrid	0.5065	0.39818	0.414	−0.441	1.4539
		Virtual	−0.6	0.45195	0.383	−1.6754	0.4754
	Virtual	Hybrid	1.1065 *	0.32785	0.003	0.3264	1.8866
		Traditional	0.6	0.45195	0.383	−0.4754	1.6754
Scheffe	Hybrid	Traditional	−0.5065	0.39818	0.448	−1.496	0.4831
		Virtual	−1.1065 *	0.32785	0.005	−1.9212	−0.2917
	Traditional	Hybrid	0.5065	0.39818	0.448	−0.4831	1.496
		Virtual	−0.6	0.45195	0.417	−1.7232	0.5232
	Virtual	Hybrid	1.1065 *	0.32785	0.005	0.2917	1.9212
		Traditional	0.6	0.45195	0.417	−0.5232	1.7232

Based on observed means. The error term is Mean Square (Error) = 1.915. * The mean difference is significant at the 0.05 level.
